# Using the safety scissors: DNA resection regulation at DNA double-strand breaks and telomeres

**DOI:** 10.1016/j.dnarep.2025.103876

**Published:** 2025-07-23

**Authors:** Michael M. Soniat, Logan R. Myler

**Affiliations:** a Department of Biochemistry and Molecular Biology, Thomas Jefferson University, Philadelphia, PA, USA; b UPMC Hillman Cancer Center, Pittsburgh, PA, USA; c Department of Pharmacology and Chemical Biology, University of Pittsburgh School of Medicine, Pittsburgh, PA, USA

**Keywords:** DNA resection, DNA double-strand break, Telomere, DNA repair, Homologous recombination, Alternative end-joining, T-loops

## Abstract

DNA resection is a universal process in genome maintenance by which one strand of DNA is degraded, leaving the other strand intact. This sometimes highly processive process is critical for many forms of DNA damage repair, replication-coupled repair, meiotic recombination, and telomere maintenance. Therefore, resection must be tightly regulated to prevent genome instability and promote faithful and accurate repair. Here, we review what is known about how resection functions and how it is controlled, using DNA double-strand break repair and telomere maintenance as examples. We address how resection is regulated in three independent steps: resection initiation, long-range processing, and termination. By addressing these mechanisms in the context of both pathways, we attempt to provide an overview of the similarities as well as the outstanding questions regarding how this robust process is regulated.

## 1. Introduction

**Fig. 1.**
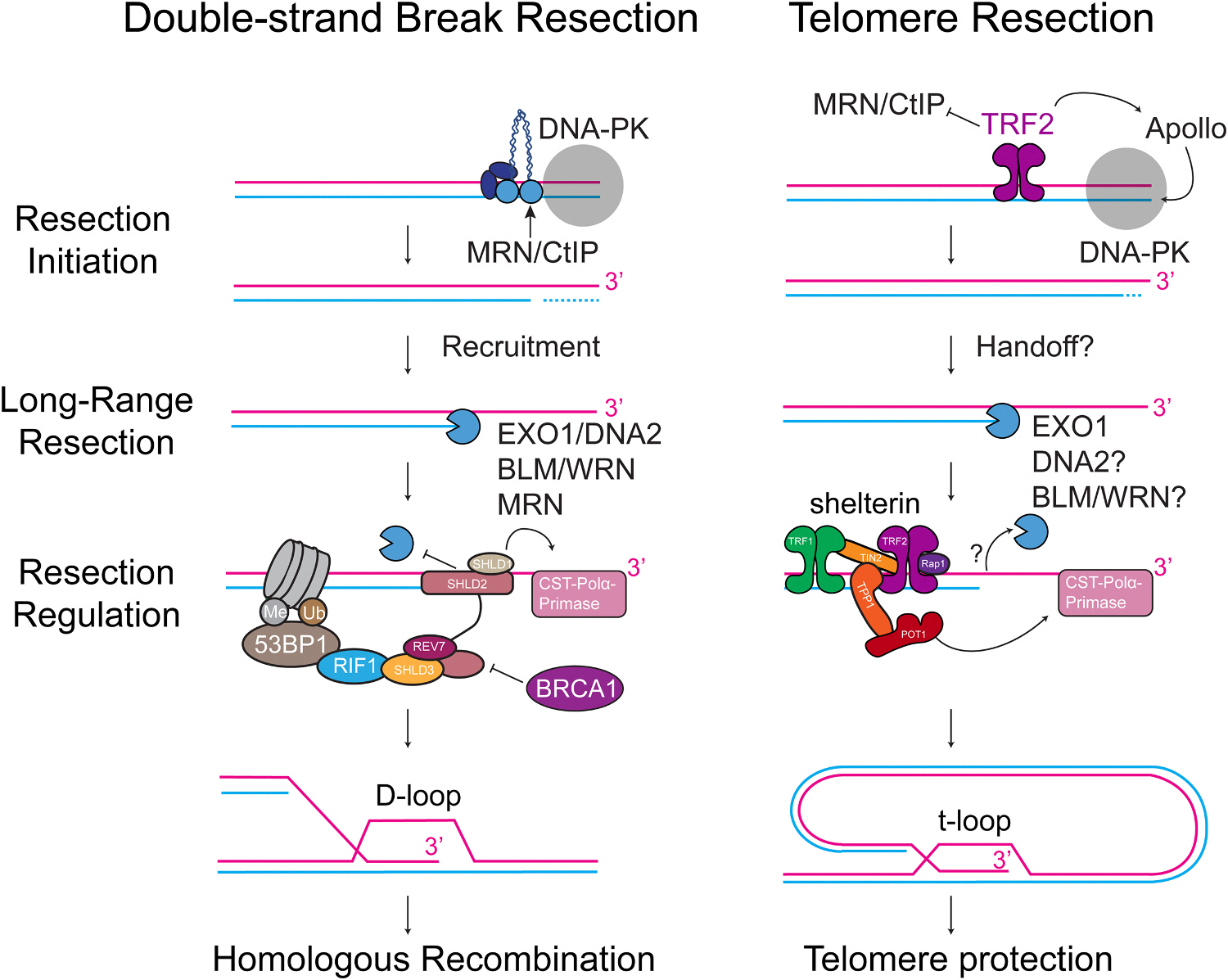
Overview of DNA double-strand break and telomere resection. (Left) Resection at DNA double-strand breaks initiates with MRN/CtIP, which nicks the DNA and recruits the resectosome, a molecular machine that mediates long-range resection. Resection is regulated by a number of factors reviewed here. Following resection, the ssDNA invades into a sister chromatid through the formation of a D-loop for downstream homology-directed repair. (Right) Telomere resection initiates with the recruitment of the nuclease Apollo by TRF2 and DNA-PK. Apollo processes a short stretch of ssDNA before handing off to the long-range nuclease EXO1 as well as other factors for further telomere processing. Resection is regulated by the telomere-binding shelterin complex, and some of the resected DNA is filled in by CST-Polα-primase, leaving a short 3’ overhang to form the protective t-loop structure.

DNA repair often necessitates the resection (digestion of the DNA; from the Latin “to cut off”) and resynthesis of specific regions of the genome in order to ensure faithful and accurate restoration of the genetic material following damage [1,2]. This resection can be performed by a variety of different nucleases in various pathways to endonucleolytically cleave the phosphate backbone of a strand of DNA or exonucleolytically digest a strand, a single nucleotide at a time, to generate long single-stranded DNA (ssDNA) [3]. One of the most common examples of the utilization of DNA resection is during the homologous recombination (HR) pathway of DNA double-strand break (DSB) repair, in which the 5’ end of a break is digested to reveal a long 3’ ssDNA overhang (Fig. 1) [4]. This overhang can be bound by recombinases such as RAD51 in human cells and utilized for invasion into a homologous sequence to resynthesize the missing DNA at a DNA break site. DNA resection is a highly conserved pathway from bacteria to humans [5]. Along with the repair of DSBs, DNA resection is a critical step in various other genome maintenance pathways, including mismatch repair, meiotic crossovers, restart of replication forks stalled at DNA lesions, and telomere end processing [6].

This review focuses on the current understanding of the mammalian DNA resection machinery and its regulation at both DSBs and telomere ends, highlighting the similarities and differences between these two processes, which are critical for maintaining genome stability.

## 2. DNA resection initiation

### 2.1. Resection initiation at DSBs

**Fig. 2.**
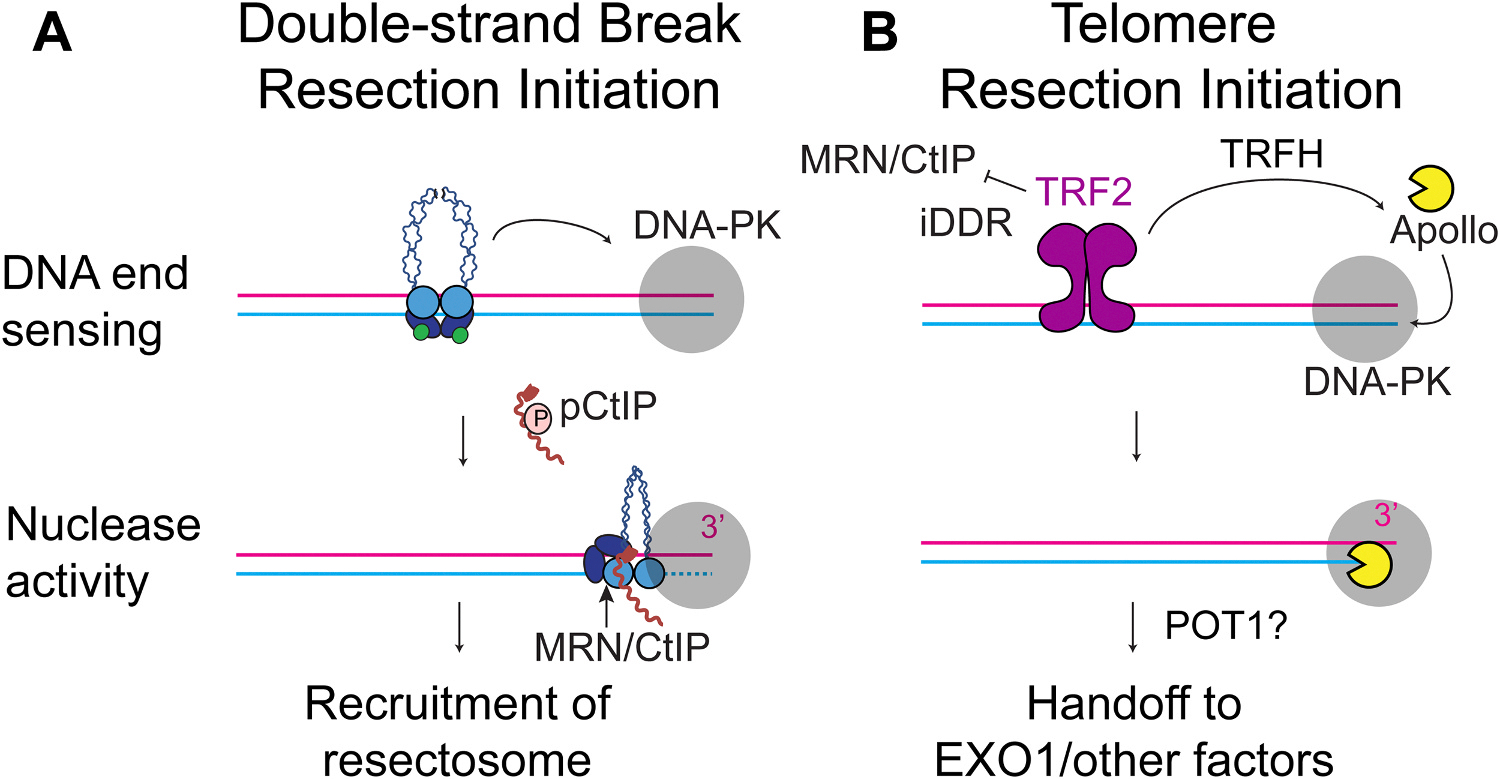
Short-range DNA resection machinery. (A) During DNA double-strand break repair initiation, the MRN complex slides to DNA ends that are already bound by the DNA-PK complex. Then, in the S/G2 phase of the cell cycle, phosphorylated CtIP activates the MRN complex to cut the 5’ end, creating a nick for loading of the long-range DNA resection machinery. (B) During telomere resection initiation, the TRF2 complex blocks MRN/CtIP cutting via its iDDR domain and instead recruits Apollo through its TRFH domain with the help of the DNA-PK complex. Apollo initially digests a short segment of the 5’ end before handing off to EXO1 and potentially other resection factors.

Resection at DSBs is initiated by the combined action of the Mre11-Rad50-Nbs1 (MRN) complex with its cofactor CtIP and represents one of the first control steps of HR (Fig. 2A). MRN locates DSBs by limited diffusion and recognizes the ends through a topological mechanism that enables even DNA ends bound by other proteins to be recognized [7,8]. Resection is limited to the S/G2 phase of the cell cycle, consistent with HR, and is triggered by the phosphorylation of CtIP by CDK2 and ATR (or potentially ATM) kinases on T847 and T859, respectively [9,10]. Phosphorylation of these two residues enables the binding of CtIP to a β-sheet surface of Rad50 (called the S-site), originally described as the locus of separation-of-function mutants in yeast (Rad50S) [11]. Rad50S mutants were capable of initiating DNA resection at mitotic DSBs in yeast but were incapable of promoting sporulation in meiosis [11]. Sporulation requires the targeted formation of DSBs by Spo11 through the formation of a protein-DNA crosslink, followed by its removal from the ends of the DNA, suggesting that a defect in initiating resection from Spo11-blocked ends is likely the reason for the sporulation defect.

MRN and CtIP initiate resection by cleaving the 5’ end of the DNA break and then loading long-range nucleases and helicases onto the nicked DNA [12]. MRN endonuclease activity cuts next to various proteins bound on the ends, including Ku, DNA-PKcs, Spo11, and TopoII. *In vitro*, MRN cuts a specific distance from the end, determined by the size of the bound protein. Biotinylated ends bound by streptavidin are cleaved ~15 nt from the end, whereas Ku and DNA-PK cleavage products are digested at 30 and 45 nt from the end, respectively, suggesting that a wide variety of blocked ends could be accessed [13].

### 2.2. Resection initiation at telomeres

Telomere ends resemble DSBs and must be protected from being recognized as damage to prevent telomere fusions or illegitimate HR. Telomere integrity is maintained by shelterin, the six-subunit telomere-binding protein complex (TRF1, TRF2, RAP1, TIN2, TPP1, POT1), and through the formation of the large lariat t-loop structure, which prevents recognition by the DSB repair machinery (Fig. 1) [14–16]. Telomere length is maintained by telomerase (a reverse transcriptase that adds telomeric sequence to the 3’ end of the telomere) as well as the ssDNA-binding complex CTC1-STN1-TEN1 (CST) and polymerase α-primase (Polα-primase) that fills in the opposite strand [17]. Following replication through telomeres, the strand replicated by leading-strand synthesis (also called the leading end) becomes blunt and lacks the 3’ overhang necessary for t-loop formation, end protection, and telomerase activity. Therefore, resection is required to digest the 5’ end and reveal the 3’ overhang. This process begins with the binding of DNA-PK to the ends and the coordinated recruitment of a 5’-3’ exonuclease, Apollo, by TRF2 and DNA-PK to digest the ends (Fig. 2B) [18–21]. Without Apollo or upon mutation of the TRFH domain of TRF2, the leading ends are not resected and fuse to other leading ends at a low frequency via the alternative-end joining pathway of DSB repair (Alt-EJ or MMEJ) [18]. Despite the similarities to DSB resection (See Fig. 1), members of the MRN complex have been shown to have no role in this process. The reasons are unclear, but recent data suggest that the iDDR domain of TRF2 regulates the MRN nuclease activity at telomeres [18, 22]. The nuclease activity of MRN is required to remove DNA-PK bound at the telomere ends, so removal of either DNA-PK or the TRF2 iDDR domain restores telomere resection even in the absence of Apollo [18]. The physiological reason for blocking MRN resection of these ends is unknown, but one might speculate that MRN-initiated resection might cause a telomere shortening defect due to the ability of MRN to completely remove DNA-PK bound to short fragments of DNA [23]. Following short-range Apollo resection, there is a hand-off to the long-range resection machinery that enables further telomere processing to generate the 3’ overhang substrate needed for telomerase activity and t-loop formation.

## 3. Long-range resection

### 3.1. Long-range resection at DSBs

**Fig. 3.**
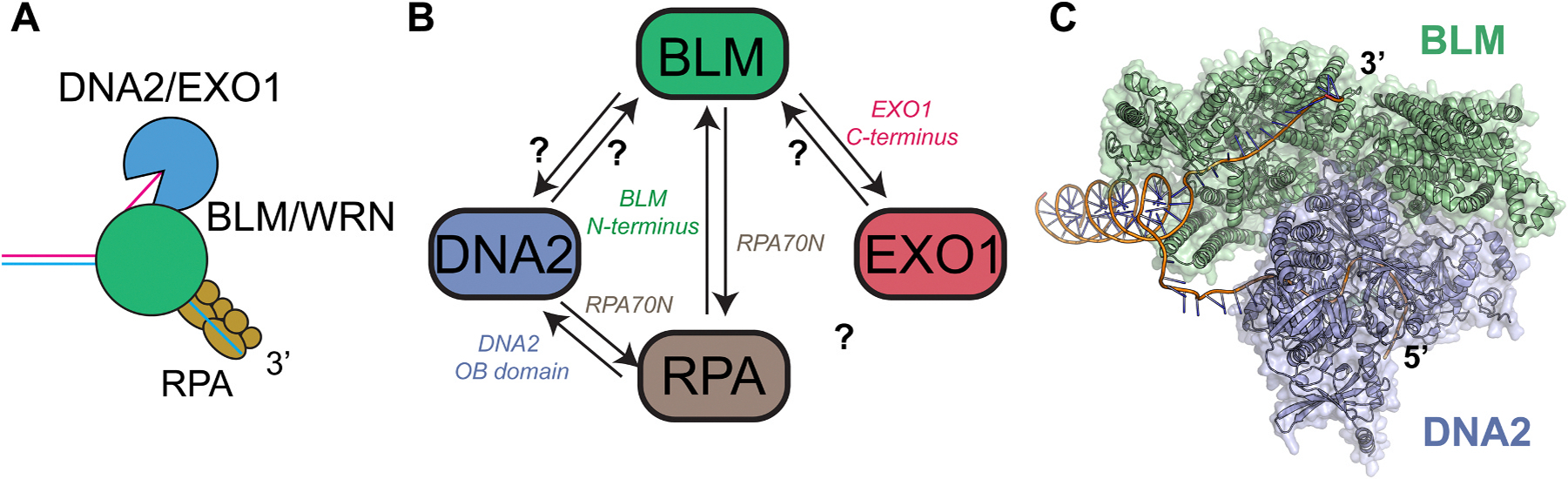
Long-range DNA resection machinery. (A) Schematic and (B) known interactions between the human DNA resection machinery. (C) AlphaFold3 analysis of the BLM-DNA2 complex during DNA resection.

Following short-range DNA resection, the MRN complex recruits a large molecular machine called the resectosome, a complex made up of a helicase - Bloom Syndrome Helicase (BLM) or Werner Syndrome Helicase (WRN), a nuclease – Exonuclease 1 (EXO1) or DNA replication ATP-dependent helicase/nuclease 2 (DNA2), as well as the ssDNA-binding protein replication protein A (RPA) and other factors (Fig. 3) [24]. The resectosome carries out long-range, exonucleolytic resection of the 5’ strand, creating a kilobase-long 3’ overhang coated by RPA [25,26]. Whereas short-range DNA resection ranges from tens to hundreds of nucleotides, long-range DNA resection can resect up to 3.5 kb from the DNA break in humans [27,28]. DNA resection is a critical step in DSB repair pathway choice, and the extent of resection can impact the fidelity of repair by mediating the balance between relatively error-free canonical HR or synthesis-dependent strand annealing (SDSA) versus error-prone Alt-EJ, single-stranded annealing (SSA), or break-induced replication (BIR) [3]. In the later stages of HR, RPA is replaced by the DNA recombinase RAD51, which catalyzes the invasion of this 3’ overhang into a sister chromatid to be used as a template for DNA synthesis. Due to the requirement of a sister chromatid for repair, HR is active primarily during the S/G2 phase of the cell cycle following DNA replication.

BLM and WRN belong to the RecQ DNA helicase family found in humans, which contains 3’-5’ helicase activity using conserved RecA-like domains [29–31]. However, along with its helicase activity, WRN is the only human RecQ helicase containing an exonuclease domain at its amino terminus [32,33]. RPA is a heterotrimeric ssDNA-binding complex comprised of the RP70, RP32, and RPA14 subunits that binds ssDNA with sub-nanomolar affinity [34,35]. Both BLM and WRN physically interact with the N-terminal OB domain of the RPA70 subunit in RPA, and RPA stimulates their helicase activity [36–39]. DNA2 is an ATP-dependent helicase/endonuclease that contains both nuclease and helicase domains [40,41]. However, DNA2 requires the helicase activity of BLM or WRN to process DNA because the helicase domain of DNA2 is not able to unwind dsDNA [42–44]. The structure of mouse DNA2 reveals that the helicase domain of DNA2 acts as a translocase in the 5’-3’ direction, threading ssDNA through a narrow protein channel requiring >10 nt to interact with both the nuclease and helicase domains [41]. Without RPA, DNA2 can digest 5’ and 3’ flaps *in vitro*; however, RPA specifically stimulates DNA2’s nuclease activity on the 5’ strand during DNA resection [45–48].

EXO1 belongs to the Rad2/XPG family of Mg^2+^-dependent nucleases, containing 5’-3’ exonuclease and 5’ flap endonuclease activities [49–51]. Unlike DNA2, EXO1 is a processive enzyme that can digest >6 kb of DNA without the aid of additional proteins [52]. EXO1 activity is tightly regulated by RPA, which strips EXO1 from DNA, promoting a distributive, multi-turnover mechanism. Previous studies have shown that BLM interacts with EXO1 and stimulates its nuclease activity in the presence of RPA [53–55].

Multiple DNA repair factors stimulate the DNA resection machinery. Along with its nuclease roles, MRN/CtIP has been shown to stimulate multiple components of the resectosome through nuclease-independent roles in yeast and humans [7,46,54,56–62]. For example, MRN has been shown to play a scaffolding role in assembling the resectosome at DSBs and melting DNA ends to promote long-range resection initiation [57]. Furthermore, MRN, through the MRE11 and NBS1 subunits, interacts with and stimulates EXO1, acting as a processivity factor in the presence of RPA [7]. MRN and CtIP also stimulate the helicase activity of BLM and DNA2’s translocase activity, respectively [56,63–65]. The BRCA1–BARD1 complex, an E3 ubiquitin ligase that localizes to DSBs via recognition of ubiquitination of H2AK15 and unmethylated H4K20, is also a major regulator of the DNA resection machinery [66]. Recent studies showed that BRCA1-BARD1 promotes both DNA resection pathways through physically interacting with the DNA helicases (BLM and WRN) and EXO1, along with stimulating both the DNA unwinding activity of BLM and WRN and the nuclease activity of EXO1 [67,68].

To date, the reason why multiple helicases and nucleases are required for DNA resection remains unknown. For example, what dictates the choice between the two RecQ helicases, or do they function redundantly? EXO1 has been suggested to be the main nuclease involved in DNA resection because the knockdown of EXO1 results in a more significant DNA resection defect than the knockdown of DNA2 [27,52, 69]. However, super-resolution microscopy studies showed that EXO1 and DNA2 are recruited and co-localized at the same DNA damage sites [70]. Additionally, recent studies have suggested that the nucleases may be required to process different DNA lesions during DNA resection [71]. For example, EXO1 could process short stretches of ribonucleotides at 5’ DNA ends, while BLM-DNA2 could process DNA substrates containing apurinic/apyrimidinic (AP) sites and oxidized bases. These results suggest that the resectosome may act as a Swiss Army knife, requiring different helicases/nucleases to process distinct barriers that the DNA resection machinery might encounter during DNA processing.

Along with HR, both Alt-EJ and SSA repair pathways of DSBs require resection to mediate homology pairing [72,73]. However, in contrast to HR, these pathways are highly mutagenic. Alt-EJ utilizes short resection lengths of 5–25 nucleotides to mediate homology pairing, often resulting in 3’ flaps that must be cleaved [74]. SSA involves DNA resection lengths of multiple kilobases, which can result in large genomic deletions [75]. Though resection length is thought to contribute to DNA repair pathway choice and fidelity, the exact mechanisms that control the choice between Alt-EJ, SSA, and HR are not fully understood.

### 3.2. Long-range resection at telomeres

Following the nucleolytic activity of the nuclease Apollo on the leading-end, both the leading-end and lagging-end telomere strands are resected by EXO1 to generate highly extended transient overhangs that are returned to a shorter length before G1 via fill-in DNA synthesis mediated by the CST-Polα-primase complex [22,76]. This process ensures that both sister telomeres obtain an overhang for t-loop formation. Telomeric DNA resection contributes to the telomere shortening rate that occurs with every round of DNA replication and, in combination with incomplete replication due to lagging-strand DNA synthesis, contributes to the end-replication problem [17]. Excessive resection can lead to shortened telomeres and premature cellular senescence activation if not tightly regulated. Many key DNA resection proteins have been shown to have similar regulatory roles in maintaining telomeric integrity in both humans and mice [77]. However, the mechanisms by which human shelterin regulates the DNA resection machinery during telomere end processing remains incomplete. For example, previous studies showed that BLM helicase alone does not play a role in telomere processing in mouse cells; however, whether the RecQ helicases (BLM and WRN) or DNA2 play a role in human telomere resection remains to be seen [22].

## 4. Regulation and outcomes of DNA resection

**Fig. 4.**
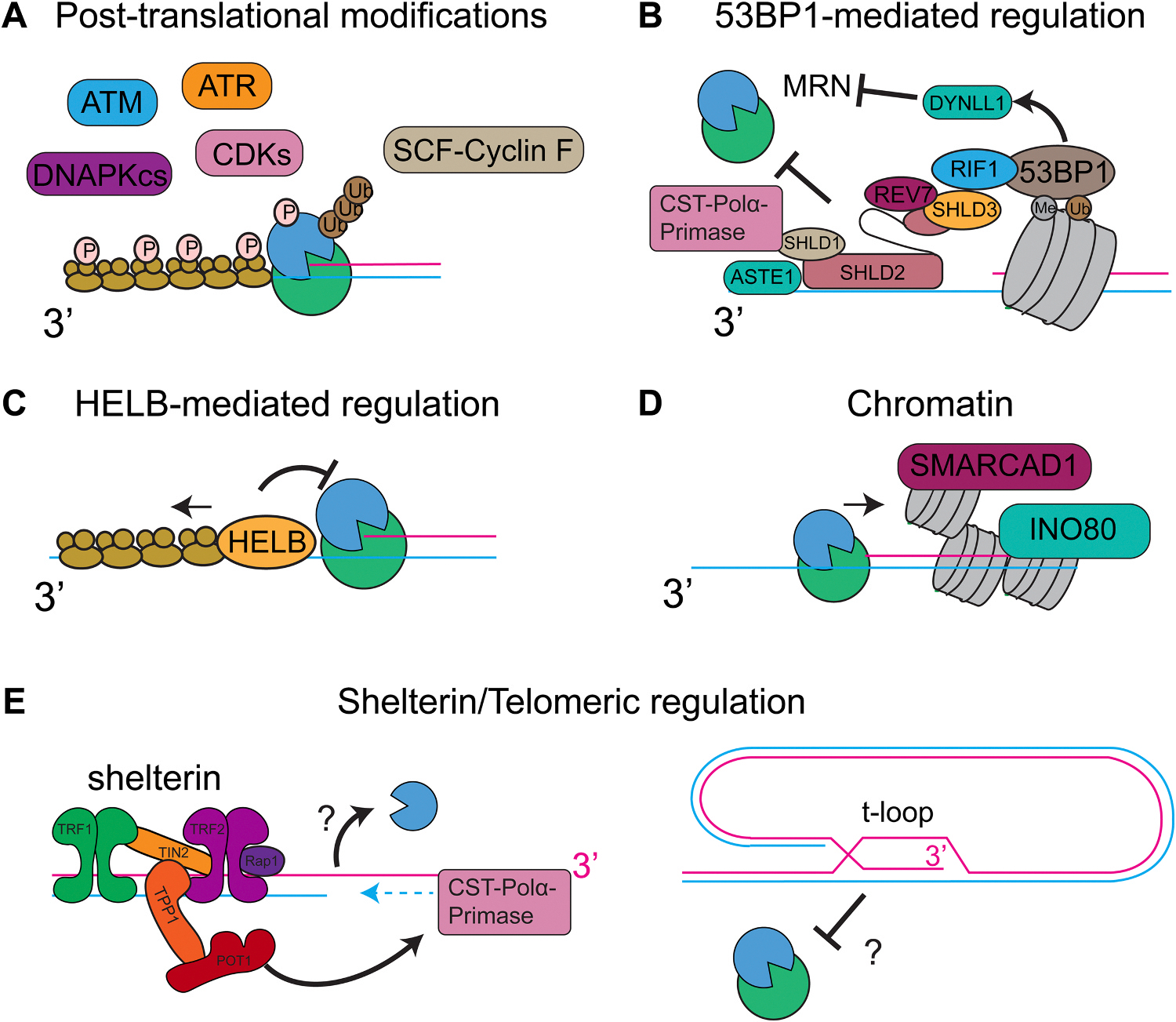
Regulatory mechanisms of long-range DNA resection Schematic of regulation of the DNA resection machinery at DNA double-strand breaks through (A) post-translational modifications, (B) the 53BP1-mediated regulators, (C) the HELB helicase, and (D) chromatin along with chromatin remodelling enzymes. (E) At telomeres, regulation of the DNA resection machinery is mediated through shelterin and the CST-Polα-primase complex or through t-loops.

Resection acts to reveal ssDNA that can be used for various recombination and annealing activities, including D-loop formation during HR and t-loop formation at telomeres. Additionally, DNA resection has been shown to aid in mobility for homology search across the genome [78–81]. Nevertheless, the length of resection needed for these structures varies. Regulation of DNA resection is critical for cell survival, where over-resection due to the lack of a homologous sequence for repair can lead to loss of genetic material and cell death. Hyper-resection can lead to the exhaustion of nuclear RPA, leading to unprotected ssDNA, which is prone to breakage and could lead to loss of genetic material. Previous studies have shown that as little as 20 bp of resected DNA is needed for HR in yeast [82]. However, longer resection tracts are needed when homologous DNA is farther from the DNA break. Additionally, limited DNA resection can occur outside of the S/G2 phase, promoting repair pathways such as Alt-EJ. These DSB pathways require the prevention of extensive long-range DNA resection. In this section, we discuss the regulators of the DNA resection machinery (Fig. 4).

### 4.1. Post-translation modifications

Many resectosome components are modified by post-translational modifications, including phosphorylation, ubiquitination, sumoylation, acetylation, and PARylation, but a unifying theme for how these modifications affect the resection machinery has not been worked out (Fig. 4A). Cyclin-dependent kinases (CDKs) phosphorylate EXO1, DNA2, and MRN in a cell-cycle-dependent manner to promote recruitment to DNA lesions during the S/G2 phase [83–85]. Phosphorylation of EXO1 by DNA damage response kinases (ATR) targets it for ubiquitination by the Skp1-Cullin1-F-box family of ubiquitin ligases and EXO1 degradation [86]. Additionally, PARP1 inhibits EXO1-mediated DNA resection [87]. In *Saccharomyces cerevisiae*, Dna2 is phosphorylated by Cdk1, which aids in Dna2 recruitment to DNA breaks and downstream resection [84]. Phosphorylation, ubiquitination, and SUMOylation have been identified in regulating BLM’s subcellular localization and mediating protein-protein interactions; however, their effect on BLM-mediated DNA resection remains unknown [88]. Additionally, ATM phosphorylates WRN to promote long-range resection [89]. RPA-ssDNA filaments activate the DNA damage response kinases that phosphorylate RPA on the N-terminus of the RPA32 subunit by multiple kinases, including ATR, DNAPKcs, and CDKs [26,90–92]. The N-terminal phosphorylation of RPA32 completely arrests DNA resection at nucleosomes via the regulation of BLM helicase [53]. This regulation depends on the interaction between the N-terminus of RPA70 and BLM.

### 4.2. 53BP1-mediated regulation

Controlling DNA resection is critical in deciding the DNA repair pathway choice. HR and the NHEJ machinery compete for the same DNA break and are mediated by the competition between the BRCA1 and 53BP1 complexes [93–97]. Until recently, the initiation of DNA resection had been considered to commit cells to HR, thus preventing repair by the NHEJ machinery. 53BP1 is recruited to DNA break through the recognition of ubiquitinated H2AK15, dimethylated H4K20, and phosphorylated H2AX (γH2AX) [98–100]. To control the DNA resection machinery, 53BP1 recruits downstream factors Replication Timing Regulatory Factor 1 (RIF1) and Pax transactivation domain interacting protein (PTIP) via phosphorylation of 53BP1 by ATM at multiple S/T-Q sites on 53BP1’s N-terminus [101,102]. PTIP has been suggested to preferentially inhibit the DNA2-mediated DNA resection pathway, whereas RIF1 inhibits the EXO1-mediated DNA resection pathway [94]. PTIP has been shown to recruit the nuclease Artemis and trim DNA ends, promoting NHEJ [103]. In contrast, it has been proposed that RIF1 inhibits DNA resection by forming higher-order compact chromatin [104]. This is supported by the X-ray crystal structure of the N-terminus of the yeast Rif1, which showed that Rif1 forms a shepherd’s crook structure wrapping DNA [105]. Furthermore, recent studies showed that RIF1 recruits the histone chaperone ASF1, which promotes chromatin compaction, inhibiting extensive DNA resection [106]. 53BP1 also recruits other major DNA resection regulators, such as the protein phosphatase 1 (PP1), dynein light chain LC8 type 1 (DYNLL1), and the shieldin complex (Fig. 4B) [107–109]. DYNLL1 physically interacts with MRE11 to disrupt the formation of the MRE11 dimer and inhibit DNA resection *in vitro* mediated by the MRN-BLM-DNA2-RPA complex [109, 110]. DYNLL1 also promotes 53BP1 oligomerization at sites of DSBs [111].

The shieldin complex is recruited by the 53BP1-RIF1 complex and is comprised of four proteins (SHLD3, REV7, SHLD2, SHLD1), which aid in the recruitment and loading of CST-Polα-primase to fill-in resected ends [112–122]. Loss of any shieldin component leads to resistance to current cancer therapeutics for BRCA-deficient and HR-deficient cancers, such as PARP inhibitors. However, a critical unresolved question is how the DNA resection machinery is disassembled to terminate DNA processing. The observation that shieldin requires Polα-dependent DNA synthesis raises questions about why ssDNA is critical for DNA end protection and the minimum length of the 3′ overhang required to inhibit HR and promote NHEJ. None of the shieldin subunits have enzymatic activity, and only two subunits, SHLD2 and SHLD3, have DNA-binding activity [116,118,123]. This has led to the hypothesis that CST is the primary inhibitor of DNA resection, which is supported by recent work showing that CST directly inhibits both EXO1 and BLM/DNA2 resection pathways[124]. Recent studies have also demonstrated that the shieldin complex recruits a DNA endonuclease, ASTE1, which is able to cleave 3’-ssDNA overhangs at DNA breaks to further inhibit the DNA resection machinery [125]. Along with the shieldin complex, DNA helicase B (HELB) has been shown to inhibit the BLM-DNA2 and EXO1-mediated DNA resection (Fig. 4C) [126]. HELB translocates on ssDNA in the 5’-3’ direction, which has led to the proposed mechanism that HELB mediates RPA removal to inhibit DNA resection [127]. However, what prevents RPA from re-binding or the exact mechanism of HELB remains to be determined.

### 4.3. Chromatin

DNA resection must occur on a highly crowded genome coated with nucleosomes and other protein roadblocks (Fig. 4D) [128]. However, how DNA resection functions in the context of chromatin is still not completely understood. Previous *in vitro* studies showed that nucleosomes strongly inhibit the EXO1 nuclease alone, though the addition of the BLM helicase stimulated both EXO1 and DNA2-mediated DNA resection past single nucleosomes [53,129]. However, at higher densities of nucleosomes, the DNA resection machinery stalled. Furthermore, nucleosomes encountered by the resectosomes were pushed along DNA without disassembling the histone octamer, suggesting other mechanisms are required to evict the nucleosome. SMARCAD1, SWI/SNF, and INO80 can evict positioned nucleosomes in a region around a broken DNA end and have been proposed to promote DNA resection [130–134]. Previous studies showed that ubiquitination of H2A by BRCA1-BARD1 recruits SMARCAD1, promoting DNA resection via repositioning or evicting nucleosomes bound by 53BP1 and its downstream factors [135]. However, the underlying mechanisms of how SMARCAD1 and INO80 promote DNA resection remain controversial because it is difficult to determine whether nucleosome eviction occurs before or as a result of ssDNA production [136]. In addition to chromatin remodelers, recent work has also implicated the incorporation of histone variants at DNA breaks, such as the histone H2A variants H2A.Z and H2A.X, in regulating DNA resection [137]. For example, H2A. Z-containing nucleosomes were shown to stimulate EXO1-mediated resection [129].

### 4.4. Telomere and shelterin-mediated regulation

Excessive DNA resection at the telomeres is a threat to telomere integrity. Disruption of the telomere fill-in machinery through POT1 or CST mutations leads to excessively long 3’ overhangs and chromosomal instability [138]. However, it is also characterized by stochastic telomere loss, the mechanism of which is unclear. Since EXO1 is processive *in vitro* and can process several kilobases of DNA, infrequent uncontrolled resection may cause severely truncated telomeres, which could be overcome by the expression of telomerase.

At deprotected telomeres, DNA resection activates the DNA damage response kinases ATM and ATR, leading to 53BP1 accumulation [139]. 53BP1 then inhibits DNA resection at telomeres via the RIF1-shieldin-CST axis, even in the absence of the shelterin complex. Similar to DSBs, this process requires the fill-in DNA synthesis of resected telomere ends by CST-Polα-primase [140–142]. Shelterin controls this hyper-resection via inhibition of ATM and ATR activation [22, 139]. TRF2 blocks activation of ATM, whereas TPP1/POT1 inhibits ATR activation [143]. TRF2 directly regulates the MRN complex activation and is also proposed to inhibit DNA resection through t-loop formation. In mice, loss of POT1a/b or TPP1 results in hyper-resection and ATR signaling; this is further exacerbated with the removal of 53BP1-RIF1 regulation. Collectively, the shelterin complex controls DNA resection in a similar manner to DSBs in order to limit ssDNA accumulation and protect genome stability (Fig. 4E).

### 4.5. DNA resection in human disease

Mutations that alter DNA resection mechanisms lead to human diseases characterized by cancer predisposition and accelerated aging, especially at the telomeres [144–146]. Defects in the telomeric fill-in machinery that lead to excessive ssDNA accumulation at telomeres cause telomere shortening, leading to telomere biology disorders such as Dyskeratosis congenita and Coats plus syndrome. Coats plus can also be caused by a mutant of POT1 (S322L in humans) that is defective in the recruitment of the CST complex [147]. Additionally, the outcomes of DNA resection and HR are becoming critical biomarkers for cancer therapy. Treatments with poly(ADP-ribose) polymerase (PARP) inhibitors have shown success in cancers that are HR-deficient, such as BRCA-deficient ovarian, breast, pancreatic, and prostate cancer, which has led to the approval of four different PARP inhibitors and many others still in clinical trials [148]. However, most patients obtain resistance to current treatments via multiple mechanisms, and current efforts are ongoing to address this challenge. A mechanism of acquired PARP inhibitor resistance is the loss of DNA resection inhibition or DNA replication fork protection factors, which results in the reacquisition of HR [149]. Therefore, there is a sparked interest in understanding the DNA resection machinery and its regulators to aid in developing new cancer therapeutics.

## 5. Concluding remarks and future directions

Our understanding of the DNA resection machinery from bacteria to humans has advanced considerably due to the advancement of cryo-electron microscopy (cryo-EM), single-molecule microscopy techniques, and next-generation sequencing, which has allowed us to monitor DNA resection at the nucleotide level both *in vitro* and in cells. However, several mechanistic questions remain unanswered. For example, why are multiple helicases and nucleases required for DNA resection, and whether specific cellular processes, such as t-loop formation or fork reversal, require a specific helicase-nuclease pair remains a central unanswered question. Furthermore, there have been advancements in understanding DNA repair mechanisms at the atomic level due to the advent of cryo-EM. However, to date, there are still limited structures of the resectosome components on DNA or in complexes with other members, possibly due to inherent flexibility in these complexes. The development of AlphaFold may allow these studies to advance faster to fully understand how the DNA resection machinery coordinates its activities to process DNA (Fig. 3C).[150]

DNA resection is a key process in maintaining genomic integrity across various regions of the genome that encounter distinctive DNA sequences and barriers, critical for DNA repair, telomere protection, meiosis, and replication. In this review, we have focused on the current understanding of the regulatory mechanisms of the DNA resection machinery at DSBs and telomeres. However, the underlying mechanisms of regulation at replication forks and meiotic DSBs are still not well understood. Additionally, we still lack a deep understanding of how resection processes DNA in the context of chromatin and how other DNA-bound barriers affect resection progression at DSBs and telomeres. Lastly, how RAD51 loading by the BRCA1-PALB2-BRCA2 complex is coordinated with DNA processing resectosome remains unknown, and deciphering the underlying mechanisms of DNA resection termination is an active field of investigation.

## References

[1] Lombard DB, Chua KF, Mostoslavsky R, Franco S, Gostissa M, Alt FW, DNA repair, genome stability, and aging, Cell 120 (2005) 497–512, 10.1016/j.cell.2005.01.028. 15734682

[2] Jeggo PA, Pearl LH, Carr AM, DNA repair, genome stability and cancer: a historical perspective, Nat. Rev. Cancer 16 (2015) 35–42, 10.1038/nrc.2015.4. 26667849

[3] Cejka P, Symington LS, DNA end resection: mechanism and control, Annu. Rev. Genet. (2021), 10.1146/annurev-genet-071719-020312. 34813349

[4] Ranjha L, Howard SM, Cejka P, Main steps in DNA double-strand break repair: an introduction to homologous recombination and related processes, Chromosoma 127 (2018) 187–214, 10.1007/s00412-017-0658-1. 29327130

[5] Wigley DB, Bacterial DNA repair: recent insights into the mechanism of RecBCD, AddAB and AdnAB, Nat. Rev. Microbiol. 11 (2012) 9–13, 10.1038/nrmicro2917. 23202527

[6] Ceccaldi R, Cejka P, Mechanisms and regulation of DNA end resection in the maintenance of genome stability, Nat. Rev. Mol. Cell Biol. (2025) 1–14, 10.1038/s41580-025-00841-4. 40133633

[7] Myler LR, Gallardo IF, Soniat MM, Deshpande RA, Gonzalez XB, Kim Y, Paull TT, Finkelstein IJ, Single-molecule imaging reveals how Mre11-Rad50-Nbs1 initiates DNA break repair, Mol. Cell 67 (2017) 891–898.e4, 10.1016/j.molcel.2017.08.002. PMC5609712 28867292

[8] Käshammer L, Saathoff J-H, Lammens K, Gut F, Bartho J, Alt A, Kessler B, Hopfner K-P, Mechanism of DNA end sensing and processing by the Mre11-Rad50 complex, Mol. Cell 76 (2019) 382–394.e6, 10.1016/j.molcel.2019.07.035. 31492634

[9] Peterson SE, Li Y, Wu-Baer F, Chait BT, Baer R, Yan H, Gottesman ME, Gautier J, Activation of DSB processing requires phosphorylation of CtIP by ATR, Mol. Cell 49 (2013) 657–667, 10.1016/j.molcel.2012.11.020. PMC3582837 23273981

[10] Huertas P, Jackson SP, Human CtIP mediates cell cycle control of DNA end resection and double strand break repair, J. Biol. Chem. 284 (2009) 9558–9565, 10.1074/jbc.M808906200. PMC2666608 19202191

[11] Alani E, Padmore R, Kleckner N, Analysis of wild-type and rad50 mutants of yeast suggests an intimate relationship between meiotic chromosome synapsis and recombination, Cell 61 (1990) 419–436, 10.1016/0092-8674(90)90524-i. 2185891

[12] Shibata A, Moiani D, Arvai AS, Perry J, Harding SM, Genois M-M, Maity R, van Rossum-Fikkert S, Kertokalio A, Romoli F, Ismail A, Ismalaj E, Petricci E, Neale MJ, Bristow RG, Masson J-Y, Wyman C, Jeggo PA, Tainer JA, DNA double-strand break repair pathway choice is directed by distinct MRE11 nuclease activities, Mol. Cell 53 (2014) 7–18, 10.1016/j.molcel.2013.11.003. PMC3909494 24316220

[13] Deshpande RA, Myler LR, Soniat MM, Makharashvili N, Lee L, Lees-Miller SP, Finkelstein IJ, Paull TT, DNA-dependent protein kinase promotes DNA end processing by MRN and CtIP, Sci. Adv. 6 (2020) eaay0922, 10.1126/sciadv.aay0922. PMC6949041 31934630

[14] De Lange T, Shelterin-mediated telomere protection, Annu. Rev. Genet. 52 (2018) 223–247, 10.1146/annurev-genet-032918-021921. 30208292

[15] de Lange T, Shelterin: the protein complex that shapes and safeguards human telomeres, Genes Dev. 19 (2005) 2100–2110, 10.1101/gad.1346005. 16166375

[16] Griffith JD, Comeau L, Rosenfield S, Stansel RM, Bianchi A, Moss H, de Lange T, Mammalian telomeres end in a large duplex loop, Cell 97 (1999) 503–514, 10.1016/s0092-8674(00)80760-6. 10338214

[17] Takai H, Aria V, Borges P, Yeeles JTP, de Lange T, CST-polymerase α-primase solves a second telomere end-replication problem, Nature 627 (2024) 664–670, 10.1038/s41586-024-07137-1. PMC11160940 38418884

[18] Myler LR, Toia B, Vaughan CK, Takai K, Matei AM, Wu P, Paull TT, de Lange T, Lottersberger F, DNA-PK and the TRF2 iDDR inhibit MRN-initiated resection at leading-end telomeres, Nat. Struct. Mol. Biol. 30 (2023) 1346–1356, 10.1038/s41594-023-01072-x. PMC10497418 37653239

[19] Sonmez C, Toia B, Eickhoff P, Matei AM, El Beyrouthy M, Wallner B, Douglas ME, de Lange T, Lottersberger F, DNA-PK controls Apollo’s access to leading-end telomeres, Nucleic Acids Res 52 (2024) 4313–4327, 10.1093/nar/gkae105. PMC11077071 38407308

[20] Wu P, van Overbeek M, Rooney S, de Lange T, Apollo contributes to G overhang maintenance and protects leading-end telomeres, Mol. Cell 39 (2010) 606–617, 10.1016/j.molcel.2010.06.031. PMC2929323 20619712

[21] Lam YC, Akhter S, Gu P, Ye J, Poulet A, Giraud-Panis M-J, Bailey SM, Gilson E, Legerski RJ, Chang S, SNMIB/Apollo protects leading-strand telomeres against NHEJ-mediated repair, EMBO J. 29 (2010) 2230–2241, 10.1038/emboj.2010.58. PMC2905253 20551906

[22] Wu P, Takai H, de Lange T, Telomeric 3′ overhangs derive from resection by Exo1 and Apollo and fill-in by POT1b-associated CST, Cell 150 (2012) 39–52, 10.1016/j.cell.2012.05.026. PMC3392515 22748632

[23] Deshpande RA, Marin-Gonzalez A, Barnes HK, Woolley PR, Ha T, Paull TT, Genome-wide analysis of DNA-PK-bound MRN cleavage products supports a sequential model of DSB repair pathway choice, Nat. Commun. 14 (2023) 5759, 10.1038/s41467-023-41544-8. PMC10505227 37717054

[24] Myler LR, Finkelstein IJ, Eukaryotic resectosomes: a single-molecule perspective, Prog. Biophys. Mol. Biol. 127 (2017) 119–129, 10.1016/j.pbiomolbio.2016.08.001. PMC5290259 27498169

[25] Chen H, Lisby M, Symington LS, RPA coordinates DNA end resection and prevents formation of DNA hairpins, Mol. Cell 50 (2013) 589–600, 10.1016/j.molcel.2013.04.032. PMC3855855 23706822

[26] Binz SK, Sheehan AM, Wold MS, Replication Protein A phosphorylation and the cellular response to DNA damage, DNA Repair 3 (2004) 1015–1024, 10.1016/j.dnarep.2004.03.028. 15279788

[27] Zhou Y, Caron P, Legube G, Paull TT, Quantitation of DNA double-strand break resection intermediates in human cells, e19–e19, Nucleic Acids Res. 42 (2014), 10.1093/nar/gkt1309. PMC3919611 24362840

[28] Canela A, Sridharan S, Sciascia N, Tubbs A, Meltzer P, Sleckman BP, Nussenzweig A, DNA breaks and end resection measured genome-wide by end sequencing, Mol. Cell 63 (2016) 898–911, 10.1016/j.molcel.2016.06.034. PMC6299834 27477910

[29] Croteau DL, Popuri V, Opresko PL, Bohr VA, Human RecQ helicases in DNA repair, recombination, and replication, Annu. Rev. Biochem. 83 (2014) 519–552, 10.1146/annurev-biochem-060713-035428. PMC4586249 24606147

[30] Newman JA, Savitsky P, Allerston CK, Bizard AH, Özer Ö, Sarlós K, Liu Y, Pardon E, Steyaert J, Hickson ID, Gileadi O, Crystal structure of the Bloom’s syndrome helicase indicates a role for the HRDC domain in conformational changes, Nucleic Acids Res. 43 (2015) 5221–5235, 10.1093/nar/gkv373. PMC4446433 25901030

[31] Newman JA, Gavard AE, Lieb S, Ravichandran MC, Hauer K, Werni P, Geist L, Böttcher J, Engen JR, Rumpel K, Samwer M, Petronczki M, Gileadi O, Structure of the helicase core of Werner helicase, a key target in microsatellite instability cancers, Life Sci. Alliance 4 (2021) e202000795, 10.26508/lsa.202000795. PMC7671478 33199508

[32] Huang S, Beresten S, Li B, Oshima J, Ellis NA, Campisi J, Characterization of the human and mouse WRN 3′→5′ exonuclease, Nucleic Acids Res. 28 (2000) 2396–2405, 10.1093/nar/28.12.2396. PMC102739 10871373

[33] Perry JJP, Yannone SM, Holden LG, Hitomi C, Asaithamby A, Han S, Cooper PK, Chen DJ, Tainer JA, WRN exonuclease structure and molecular mechanism imply an editing role in DNA end processing, Nat. Struct. Mol. Biol. 13 (2006) 414–422, 10.1038/nsmb1088. 16622405

[34] Bhat KP, Cortez D, RPA and RAD51: fork reversal, fork protection, and genome stability, Nat. Struct. Mol. Biol. 25 (2018) 446–453, 10.1038/s41594-018-0075-z. PMC6006513 29807999

[35] Fan J, Pavletich NP, Structure and conformational change of a replication protein A heterotrimer bound to ssDNA, Genes Dev. 26 (2012) 2337–2347, 10.1101/gad.194787.112. PMC3475805 23070815

[36] Doherty KM, Sommers JA, Gray MD, Lee JW, von Kobbe C, Thoma NH, Kureekattil RP, Kenny MK, Brosh RM, Physical and functional mapping of the replication protein a interaction domain of the werner and bloom syndrome helicases, J. Biol. Chem. 280 (2005) 29494–29505, 10.1074/jbc.m500653200. 15965237

[37] Brosh RM, Li J-L, Kenny MK, Karow JK, Cooper MP, Kureekattil RP, Hickson ID, Bohr VA, Replication protein A physically interacts with the Bloom syndrome protein and stimulates its helicase activity, J. Biol. Chem. 275 (2000) 23500–23508, 10.1074/jbc.m001557200. 10825162

[38] Lee M, Shin S, Uhm H, Hong H, Kirk J, Hyun K, Kulikowicz T, Kim J, Ahn B, Bohr VA, Hohng S, Multiple RPAs make WRN syndrome protein a superhelicase, Nucleic Acids Res. 46 (2018) gky272, 10.1093/nar/gky272. PMC5961295 29668972

[39] Wu Y, Fu W, Zang N, Zhou C, Structural characterization of human RPA70N association with DNA damage response proteins, eLife 12 (2023), 10.7554/elife.81639. PMC10479964 37668474

[40] Symington LS, Mechanism and regulation of DNA end resection in eukaryotes, Crit. Rev. Biochem. Mol. Biol. 51 (2016) 195–212, 10.3109/10409238.2016.1172552. PMC4957645 27098756

[41] Zhou C, Pourmal S, Pavletich NP, Dna2 nuclease-helicase structure, mechanism and regulation by Rpa, Elife 4 (2015) e09832, 10.7554/eLife.09832. PMC4716839 26491943

[42] Sturzenegger A, Burdova K, Kanagaraj R, Levikova M, Pinto C, Cejka P, Janscak P, DNA2 cooperates with the WRN and BLM RecQ helicases to mediate long-range DNA end resection in human cells, J. Biol. Chem. 289 (2014) 27314–27326, 10.1074/jbc.m114.578823. PMC4175362 25122754

[43] Levikova M, Pinto C, Cejka P, The motor activity of DNA2 functions as an ssDNA translocase to promote DNA end resection, Genes Dev. 31 (2017) 493–502, 10.1101/gad.295196.116. 28336515 PMC5393063

[44] Miller AS, Daley JM, Pham NT, Niu H, Xue X, Ira G, Sung P, A novel role of the Dna2 translocase function in DNA break resection, Genes Dev. 31 (2017) 503–510, 10.1101/gad.295659.116. 28336516 PMC5393064

[45] Masuda-Sasa T, Biochemical analysis of human Dna2, Nucleic Acids Res. 34 (2006) 1865–1875, 10.1093/nar/gkl070. 16595800 PMC1428797

[46] Cejka P, Cannavo E, Polaczek P, Masuda-Sasa T, Pokharel S, Campbell JL, Kowalczykowski SC, DNA end resection by Dna2–Sgs1–RPA and its stimulation by Top3–Rmi1 and Mre11–Rad50–Xrs2, Nature 467 (2010) 112–116, 10.1038/nature09355. 20811461 PMC3089589

[47] Daley JM, Chiba T, Xue X, Niu H, Sung P, Multifaceted role of the Topo IIIα_–_RMI1-RMI2 complex and DNA2 in the BLM-dependent pathway of DNA break end resection, Nucleic Acids Res. 42 (2014) 11083–11091, 10.1093/nar/gku803. 25200081 PMC4176181

[48] Acharya A, Kasaciunaite K, Göse M, Kissling V, Guérois R, Seidel R, Cejka P, Distinct RPA domains promote recruitment and the helicase-nuclease activities of Dna2, Nat. Commun. 12 (2021) 6521, 10.1038/s41467-021-26863-y. 34764291 PMC8586334

[49] Cejka P, DNA end resection: nucleases team up with the right partners to initiate homologous recombination*, J. Biol. Chem. 290 (2015) 22931–22938, 10.1074/jbc.r115.675942. 26231213 PMC4645618

[50] Orans J, McSweeney EA, Iyer RR, Hast MA, Hellinga HW, Modrich P, Beese LS, Structures of human exonuclease 1 DNA complexes suggest a unified mechanism for nuclease family, Cell 145 (2011) 212–223, 10.1016/j.cell.2011.03.005. 21496642 PMC3093132

[51] Shi Y, Hellinga HW, Beese LS, Interplay of catalysis, fidelity, threading, and processivity in the exo- and endonucleolytic reactions of human exonuclease I, Proc. Natl. Acad. Sci. USA 114 (2017) 6010–6015, 10.1073/pnas.1704845114. 28533382 PMC5468604

[52] Myler LR, Gallardo IF, Zhou Y, Gong F, Yang S-H, Wold MS, Miller KM, Paull TT, Finkelstein IJ, Single-molecule imaging reveals the mechanism of Exo1 regulation by single-stranded DNA binding proteins, Proc. Natl. Acad. Sci. 113 (2016) E1170–E1179, 10.1073/pnas.1516674113. 26884156 PMC4780606

[53] Soniat MM, Myler LR, Kuo H-C, Paull TT, Finkelstein IJ, RPA phosphorylation inhibits DNA resection, Mol. Cell 75 (2019) 145–153.e5, 10.1016/j.molcel.2019.05.005. 31153714 PMC6625828

[54] Nimonkar AV, Genschel J, Kinoshita E, Polaczek P, Campbell JL, Wyman C, Modrich P, Kowalczykowski SC, BLM–DNA2–RPA–MRN and EXO1–BLM–RPA–MRN constitute two DNA end resection machineries for human DNA break repair, Genes Dev. 25 (2011) 350–362, 10.1101/gad.2003811. 21325134 PMC3042158

[55] Nimonkar AV, Özsoy AZ, Genschel J, Modrich P, Kowalczykowski SC, Human exonuclease 1 and BLM helicase interact to resect DNA and initiate DNA repair, Proc. Natl. Acad. Sci. USA 105 (2008) 16906–16911, 10.1073/pnas.0809380105. 18971343 PMC2579351

[56] Soniat MM, Nguyen G, Kuo H-C, Finkelstein IJ, The MRN complex and topoisomerase IIIa-RMI1/2 synchronize DNA resection motor proteins, J. Biol. Chem. 299 (2023) 102802, 10.1016/j.jbc.2022.102802. 36529288 PMC9971906

[57] Cannon B, Kuhnlein J, Yang S-H, Cheng A, Schindler D, Stark JM, Russell R, Paull TT, Visualization of local DNA unwinding by Mre11/Rad50/Nbs1 using single-molecule FRET, Proc. Natl. Acad. Sci. 110 (2013) 18868–18873, 10.1073/pnas.1309816110. 24191051 PMC3839711

[58] Niu H, Chung W-H, Zhu Z, Kwon Y, Zhao W, Chi P, Prakash R, Seong C, Liu D, Lu L, Ira G, Sung P, Mechanism of the ATP-dependent DNA end-resection machinery from Saccharomyces cerevisiae, Nature 467 (2010) 108–111, 10.1038/nature09318. 20811460 PMC2955862

[59] Zhu Z, Chung W-H, Shim EY, Lee SE, Ira G, Sgs1 Helicase and Two Nucleases Dna2 and Exo1 Resect DNA Double-Strand Break Ends, Cell 134 (2008) 981–994, 10.1016/j.cell.2008.08.037. 18805091 PMC2662516

[60] Nicolette ML, Lee K, Guo Z, Rani M, Chow JM, Lee SE, Paull TT, Mre11–Rad50–Xrs2 and Sae2 promote 5′ strand resection of DNA double-strand breaks, Nat. Struct. Mol. Biol. 17 (2010) 1478–1485, 10.1038/nsmb.1957. 21102445 PMC3059534

[61] Mimitou EP, Symington LS, Sae2, Exo1 and Sgs1 collaborate in DNA double-strand break processing, Nature 455 (2008) 770–774, 10.1038/nature07312. 18806779 PMC3818707

[62] Cannavo E, Cejka P, Kowalczykowski SC, Relationship of DNA degradation by Saccharomyces cerevisiae exonuclease 1 and its stimulation by RPA and Mre11-Rad50-Xrs2 to DNA end resection, Proc. Natl. Acad. Sci. 110 (2013) E1661–E1668, 10.1073/pnas.1305166110. 23589858 PMC3645542

[63] Ceppi I, Howard SM, Kasaciunaite K, Pinto C, Anand R, Seidel R, Cejka P, CtIP promotes the motor activity of DNA2 to accelerate long-range DNA end resection, Proc. Natl. Acad. Sci. USA 117 (2020) 8859–8869, 10.1073/pnas.2001165117. 32241893 PMC7183222

[64] Daley JM, Jimenez-Sainz J, Wang W, Miller AS, Xue X, Nguyen KA, Jensen RB, Sung P, Enhancement of BLM-DNA2-mediated long-range DNA end resection by CtIP, Cell Rep. 21 (2017) 324–332, 10.1016/j.celrep.2017.09.048. 29020620 PMC5689478

[65] Ceppi I, Cannavo E, Bret H, Camarillo R, Vivalda F, Thakur RS, Romero-Franco A, Sartori AA, Huertas P, Guérois R, Cejka P, PLK1 regulates CtIP and DNA2 interplay in long-range DNA end resection, Genes Dev. (2023), 10.1101/gad.349981.122. PMC10069449 36746606

[66] Hu Q, Botuyan MV, Zhao D, Cui G, Mer E, Mer G, Mechanisms of BRCA1–BARD1 nucleosome recognition and ubiquitylation, Nature 596 (2021) 438–443, 10.1038/s41586-021-03716-8. 34321665 PMC8680157

[67] Salunkhe S, Daley JM, Kaur H, Tomimatsu N, Xue C, Raina VB, Jasper AM, Rogers CM, Li W, Zhou S, Mojidra R, Kwon Y, Fang Q, Ji J-H, Badamchi Shabestari A, Fitzgerald O, Dinh H, Mukherjee B, Habib AA, Hromas R, Mazin AV, Wasmuth EV, Olsen SK, Libich DS, Zhou D, Zhao W, Greene EC, Burma S, Sung P, Promotion of DNA end resection by BRCA1-BARD1 in homologous recombination, Nature 634 (2024) 482–491, 10.1038/s41586-024-07910-2. 39261729 PMC11539920

[68] Ceppi I, Dello Stritto MR, Mütze M, Braunshier S, Mengoli V, Reginato G, Võ HMP, Jimeno S, Acharya A, Roy M, Sanchez A, Halder S, Howard SM, Guérois R, Huertas P, Noordermeer SM, Seidel R, Cejka P, Mechanism of BRCA1-BARD1 function in DNA end resection and DNA protection, Nature 634 (2024) 492–500, 10.1038/s41586-024-07909-9. 39261728 PMC11464378

[69] Bolderson E, Tomimatsu N, Richard DJ, Boucher D, Kumar R, Pandita TK, Burma S, Khanna KK, Phosphorylation of Exo1 modulates homologous recombination repair of DNA double-strand breaks, Nucleic Acids Res. 38 (2010) 1821–1831, 10.1093/nar/gkp1164. 20019063 PMC2847229

[70] Whelan DR, Rothenberg E, Super-resolution mapping of cellular double-strand break resection complexes during homologous recombination, Proc. Natl. Acad. Sci. 118 (2021) e2021963118, 10.1073/pnas.2021963118. 33707212 PMC7980414

[71] Daley JM, Tomimatsu N, Hooks G, Wang W, Miller AS, Xue X, Nguyen KA, Kaur H, Williamson E, Mukherjee B, Hromas R, Burma S, Sung P, Specificity of end resection pathways for double-strand break regions containing ribonucleotides and base lesions, Nat. Commun. 11 (2020) 3088, 10.1038/s41467-020-16903-4. 32555206 PMC7303207

[72] Bhargava R, Onyango DO, Stark JM, Regulation of single-strand annealing and its role in genome maintenance, Trends Genet. 32 (2016) 566–575, 10.1016/j.tig.2016.06.007. 27450436 PMC4992407

[73] Chang HHY, Pannunzio NR, Adachi N, Lieber MR, Non-homologous DNA end joining and alternative pathways to double-strand break repair, Nat. Rev. Mol. Cell Biol. 18 (2017) 495–506, 10.1038/nrm.2017.48. 28512351 PMC7062608

[74] Seol J-H, Shim EY, Lee SE, Microhomology-mediated end joining: good, bad and ugly, Mutat. Res. Fundam. Mol. Mech. Mutagen. 809 (2018) 81–87, 10.1016/j.mrfmmm.2017.07.002. PMC6477918 28754468

[75] Mendez-Dorantes C, Tsai LJ, Jahanshir E, Lopezcolorado FW, Stark JM, BLM has contrary effects on repeat-mediated deletions, based on the distance of DNA DSBs to a repeat and repeat divergence, Cell Rep. 30 (2020) 1342–1357.e4, 10.1016/j.celrep.2020.01.001. 32023454 PMC7085117

[76] Chow TT, Zhao Y, Mak SS, Shay JW, Wright WE, Early and late steps in telomere overhang processing in normal human cells: the position of the final RNA primer drives telomere shortening, Genes Dev. 26 (2012) 1167–1178, 10.1101/gad.187211.112. 22661228 PMC3371406

[77] Doksani Y, de Lange T, The role of double-strand break repair pathways at functional and dysfunctional telomeres, Cold Spring Harb. Perspect. Biol. 6 (2014) a016576, 10.1101/cshperspect.a016576. 25228584 PMC4292156

[78] Kimble MT, Johnson MJ, Nester MR, Symington LS, Long-range DNA end resection supports homologous recombination by checkpoint activation rather than extensive homology generation, eLife 12 (2023) e84322, 10.7554/elife.84322. 37387287 PMC10400078

[79] Chiolo I, Altmeyer M, Legube G, Mekhail K, Nuclear and genome dynamics underlying DNA double-strand break repair, Nat. Rev. Mol. Cell Biol. (2025) 1–20, 10.1038/s41580-025-00828-1. 40097581 PMC12282718

[80] Miné-Hattab J, Rothstein R, Increased chromosome mobility facilitates homology search during recombination, Nat. Cell Biol. 14 (2012) 510–517, 10.1038/ncb2472. 22484485

[81] Dion V, Kalck V, Horigome C, Towbin BD, Gasser SM, Increased mobility of double-strand breaks requires Mec1, Rad9 and the homologous recombination machinery, Nat. Cell Biol. 14 (2012) 502–509, 10.1038/ncb2465. 22484486

[82] Hua S, Qiu M, Chan E, Zhu L, Luo Y, Minimum length of sequence homology required for *in vivo* cloning by homologous recombination in yeast, Plasmid 38 (1997) 91–96, 10.1006/plas.1997.1305. 9339466

[83] Tomimatsu N, Mukherjee B, Hardebeck MC, Ilcheva M, Camacho CV, Harris JL, Porteus M, Llorente B, Khanna KK, Burma S, Phosphorylation of EXO1 by CDKs 1 and 2 regulates DNA end resection and repair pathway choice, Nat. Commun. 5 (2014) 3561, 10.1038/ncomms4561. 24705021 PMC4041212

[84] Chen X, Niu H, Chung W-H, Zhu Z, Papusha A, Shim EY, Lee SE, Sung P, Ira G, Cell cycle regulation of DNA double-strand break end resection by Cdk1-dependent Dna2 phosphorylation, Nat. Struct. Mol. Biol. 18 (2011) 1015–1019, 10.1038/nsmb.2105. 21841787 PMC3168961

[85] Ferretti LP, Lafranchi L, Sartori AA, Controlling DNA-end resection: a new task for CDKs, Front. Genet. 4 (2013) 99, 10.3389/fgene.2013.00099. 23760669 PMC3669801

[86] Tomimatsu N, Mukherjee B, Harris JL, Boffo FL, Hardebeck MC, Potts PR, Khanna KK, Burma S, DNA-damage-induced degradation of EXO1 exonuclease limits DNA end resection to ensure accurate DNA repair, J. Biol. Chem. 292 (2017) 10779–10790, 10.1074/jbc.m116.772475. 28515316 PMC5491765

[87] Caron M-C, Sharma AK, O’Sullivan J, Myler LR, Ferreira MT, Rodrigue A, Coulombe Y, Ethier C, Gagné J-P, Langelier M-F, Pascal JM, Finkelstein IJ, Hendzel MJ, Poirier GG, Masson J-Y, Poly(ADP-ribose) polymerase-1 antagonizes DNA resection at double-strand breaks, Nat. Commun. 10 (2019) 2954, 10.1038/s41467-019-10741-9. 31273204 PMC6609622

[88] Böhm S, Bernstein KA, The role of post-translational modifications in fine-tuning BLM helicase function during DNA repair, DNA Repair 22 (2014) 123–132, 10.1016/j.dnarep.2014.07.007. 25150915 PMC4175148

[89] Palermo V, Malacaria E, Semproni M, Camerini S, Casella M, Perdichizzi B, Valenzisi P, Sanchez M, Marini F, Pellicioli A, Franchitto A, Pichierri P, Switch-like phosphorylation of WRN integrates end-resection with RAD51 metabolism at collapsed replication forks, Nucleic Acids Res. 52 (2024) 12334–12350, 10.1093/nar/gkae807. 39315694 PMC11551760

[90] Fousek-Schuller VJ, Borgstahl GEO, The intriguing mystery of RPA phosphorylation in DNA double-strand break repair, Genes 15 (2024) 167, 10.3390/genes15020167. 38397158 PMC10888239

[91] Blackford AN, Jackson SP, ATM ATR, and DNA-PK: the trinity at the heart of the DNA damage response, Mol. Cell 66 (2017) 801–817, 10.1016/j.molcel.2017.05.015. 28622525

[92] Matsuoka S, Ballif BA, Smogorzewska A, McDonald ER, Hurov KE, Luo J, Bakalarski CE, Zhao Z, Solimini N, Lerenthal Y, Shiloh Y, Gygi SP, Elledge SJ, ATM and ATR substrate analysis reveals extensive protein networks responsive to DNA damage, Science 316 (2007) 1160–1166, 10.1126/science.1140321. 17525332

[93] Mirman Z, de Lange T, 53BP1: a DSB escort, Genes Dev. 34 (2020) 7–23, 10.1101/gad.333237.119. 31896689 PMC6938671

[94] Callen E, Zong D, Wu W, Wong N, Stanlie A, Ishikawa M, Pavani R, Dumitrache LC, Byrum AK, Mendez-Dorantes C, Martinez P, Canela A, Maman Y, Day A, Kruhlak MJ, Blasco MA, Stark JM, Mosammaparast N, McKinnon PJ, Nussenzweig A, 53BP1 enforces distinct pre- and post-resection blocks on homologous recombination, Mol. Cell 77 (2019) 26–38.e7, 10.1016/j.molcel.2019.09.024. 31653568 PMC6993210

[95] Bunting SF, Callén E, Wong N, Chen H-T, Polato F, Gunn A, Bothmer A, Feldhahn N, Fernandez-Capetillo O, Cao L, Xu X, Deng C-X, Finkel T, Nussenzweig M, Stark JM, Nussenzweig A, 53BP1 inhibits homologous recombination in Brca1-deficient cells by blocking resection of DNA breaks, Cell 141 (2010) 243–254, 10.1016/j.cell.2010.03.012. 20362325 PMC2857570

[96] Callen E, Di Virgilio M, Kruhlak MJ, Nieto-Soler M, Wong N, Chen H-T, Faryabi RB, Polato F, Santos M, Starnes LM, Wesemann DR, Lee J-E, Tubbs A, Sleckman BP, Daniel JA, Ge K, Alt FW, Fernandez-Capetillo O, Nussenzweig MC, Nussenzweig A, 53BP1 mediates productive and mutagenic DNA repair through distinct phosphoprotein interactions, Cell 153 (2013) 1266–1280, 10.1016/j.cell.2013.05.023. 23727112 PMC3713552

[97] Escribano-Díaz C, Orthwein A, Fradet-Turcotte A, Xing M, Young JTF, Tkáč J, Cook MA, Rosebrock AP, Munro M, Canny MD, Xu D, Durocher D, A cell cycle-dependent regulatory circuit composed of 53BP1-RIF1 and BRCA1-CtIP controls DNA repair pathway choice, Mol. Cell 49 (2013) 872–883, 10.1016/j.molcel.2013.01.001. 23333306

[98] Wilson MD, Benlekbir S, Fradet-Turcotte A, Sherker A, Julien J-P, McEwan A, Noordermeer SM, Sicheri F, Rubinstein JL, Durocher D, The structural basis of modified nucleosome recognition by 53BP1, Nature 536 (2016) 100–103, 10.1038/nature18951. 27462807

[99] Fradet-Turcotte A, Canny MD, Escribano-Díaz C, Orthwein A, Leung CCY, Huang H, Landry M-C, Kitevski-LeBlanc J, Noordermeer SM, Sicheri F, Durocher D, 53BP1 is a reader of the DNA-damage-induced H2A Lys 15 ubiquitin mark, Nature 499 (2013) 50–54, 10.1038/nature12318. 23760478 PMC3955401

[100] Kelliher JL, Folkerts ML, Shen KV, Song W, Tengler K, Stiefel CM, Lee S-O, Dray E, Zhao W, Koss B, Pannunzio NR, Leung JW, Evolved histone tail regulates 53BP1 recruitment at damaged chromatin, Nat. Commun. 15 (2024) 4634, 10.1038/s41467-024-49071-w. 38821984 PMC11143218

[101] Setiaputra D, Escribano-Díaz C, Reinert JK, Sadana P, Zong D, Callen E, Sifri C, Seebacher J, Nussenzweig A, Thomä NH, Durocher D, RIF1 acts in DNA repair through phosphopeptide recognition of 53BP1, Mol. Cell (2022), 10.1016/j.molcel.2022.01.025. PMC8995355 35216668

[102] Munoz IM, Jowsey PA, Toth R, Rouse J, Phospho-epitope binding by the BRCT domains of hPTIP controls multiple aspects of the cellular response to DNA damage, Nucleic Acids Res. 35 (2007) 5312–5322, 10.1093/nar/gkm493. 17690115 PMC2018624

[103] Wang J, Aroumougame A, Lobrich M, Li Y, Chen D, Chen J, Gong Z, PTIP associates with Artemis to dictate DNA repair pathway choice, Genes Dev. 28 (2014) 2693–2698, 10.1101/gad.252478.114. 25512557 PMC4265673

[104] Ochs F, Karemore G, Miron E, Brown J, Sedlackova H, Rask M-B, Lampe M, Buckle V, Schermelleh L, Lukas J, Lukas C, Stabilization of chromatin topology safeguards genome integrity, Nature 574 (2019) 571–574, 10.1038/s41586-019-1659-4. 31645724

[105] Mattarocci S, Reinert JK, Bunker RD, Fontana GA, Shi T, Klein D, Cavadini S, Faty M, Shyian M, Hafner L, Shore D, Thomä NH, Rass U, Rif1 maintains telomeres and mediates DNA repair by encasing DNA ends, Nat. Struct. Mol. Biol. 24 (2017) 588–595, 10.1038/nsmb.3420. 28604726

[106] Feng S, Ma S, Li K, Gao S, Ning S, Shang J, Guo R, Chen Y, Blumenfeld B, Simon I, Li Q, Guo R, Xu D, RIF1-ASF1-mediated high-order chromatin structure safeguards genome integrity, Nat. Commun. 13 (2022) 957, 10.1038/s41467-022-28588-y. 35177609 PMC8854732

[107] Isobe S-Y, Hiraga S, Nagao K, Sasanuma H, Donaldson AD, Obuse C, Protein phosphatase 1 acts as a RIF1 effector to suppress DSB resection prior to Shieldin action, Cell Rep. 36 (2021) 109383, 10.1016/j.celrep.2021.109383. 34260925 PMC8293623

[108] Setiaputra D, Durocher D, Shieldin – the protector of DNA ends, EMBO Rep. 20 (2019), 10.15252/embr.201847560. PMC6501030 30948458

[109] He YJ, Meghani K, Caron M-C, Yang C, Ronato DA, Bian J, Sharma A, Moore J, Niraj J, Detappe A, Doench JG, Legube G, Root DE, D’Andrea AD, Drané P, De S, Konstantinopoulos PA, Masson J-Y, Chowdhury D, DYNLL1 binds to MRE11 to limit DNA end resection in BRCA1-deficient cells, Nature 563 (2018) 522–526, 10.1038/s41586-018-0670-5. 30464262 PMC7155769

[110] Swift ML, Zhou R, Syed A, Moreau LA, Tomasik B, Tainer JA, Konstantinopoulos PA, D’Andrea AD, He YJ, Chowdhury D, Dynamics of the DYNLL1–MRE11 complex regulate DNA end resection and recruitment of Shieldin to DSBs, Nat. Struct. Mol. Biol. (2023) 1–12, 10.1038/s41594-023-01074-9. PMC10686051 37696958

[111] Becker JR, Cuella-Martin R, Barazas M, Liu R, Oliveira C, Oliver AW, Bilham K, Holt AB, Blackford AN, Heierhorst J, Jonkers J, Rottenberg S, Chapman JR, The ASCIZ-DYNLL1 axis promotes 53BP1-dependent non-homologous end joining and PARP inhibitor sensitivity, Nat. Commun. 9 (2018) 5406, 10.1038/s41467-018-07855-x. 30559443 PMC6297349

[112] Mirman Z, Lottersberger F, Takai H, Kibe T, Gong Y, Takai K, Bianchi A, Zimmermann M, Durocher D, de Lange T, 53BP1–RIF1–shieldin counteracts DSB resection through CST- and Polα-dependent fill-in, Nature 560 (2018) 112–116, 10.1038/s41586-018-0324-7. 30022158 PMC6072559

[113] Dev H, Chiang T-WW, Lescale C, de Krijger I, Martin AG, Pilger D, Coates J, Sczaniecka-Clift M, Wei W, Ostermaier M, Herzog M, Lam J, Shea A, Demir M, Wu Q, Yang F, Fu B, Lai Z, Balmus G, Belotserkovskaya R, Serra V, O’Connor MJ, Bruna A, Beli P, Pellegrini L, Caldas C, Deriano L, Jacobs JJL, Galanty Y, Jackson SP, Shieldin complex promotes DNA end-joining and counters homologous recombination in BRCA1-null cells, Nat. Cell Biol. 20 (2018) 954–965, 10.1038/s41556-018-0140-1. 30022119 PMC6145444

[114] Ghezraoui H, Oliveira C, Becker JR, Bilham K, Moralli D, Anzilotti C, Fischer R, Deobagkar-Lele M, Sanchiz-Calvo M, Fueyo-Marcos E, Bonham S, Kessler BM, Rottenberg S, Cornall RJ, Green CM, Chapman JR, 53BP1 cooperation with the REV7–shieldin complex underpins DNA structure-specific NHEJ, Nature 560 (2018) 122–127, 10.1038/s41586-018-0362-1. 30046110 PMC6989217

[115] Gupta R, Somyajit K, Narita T, Maskey E, Stanlie A, Kremer M, Typas D, Lammers M, Mailand N, Nussenzweig A, Lukas J, Choudhary C, DNA repair network analysis reveals shieldin as a key regulator of NHEJ and PARP inhibitor sensitivity, Cell 173 (2018) 972–988.e23, 10.1016/j.cell.2018.03.050. 29656893 PMC8108093

[116] Noordermeer SM, Adam S, Setiaputra D, Barazas M, Pettitt SJ, Ling AK, Olivieri M, Álvarez-Quilón A, Moatti N, Zimmermann M, Annunziato S, Krastev DB, Song F, Brandsma I, Frankum J, Brough R, Sherker A, Landry S, Szilard RK, Munro MM, McEwan A, de Rugy TG, Lin Z-Y, Hart T, Moffat J, Gingras A-C, Martin A, van Attikum H, Jonkers J, Lord CJ, Rottenberg S, Durocher D, The shieldin complex mediates 53BP1-dependent DNA repair, Nature 560 (2018) 117–121, 10.1038/s41586-018-0340-7. 30022168 PMC6141009

[117] Findlay S, Heath J, Luo VM, Malina A, Morin T, Coulombe Y, Djerir B, Li Z, Samiei A, Simo-Cheyou E, Karam M, Bagci H, Rahat D, Grapton D, Lavoie EG, Dove C, Khaled H, Kuasne H, Mann KK, Klein KO, Greenwood CM, Tabach Y, Park M, Côté J, Masson J, Maréchal A, Orthwein A, SHLD2/FAM35A co-operates with REV7 to coordinate DNA double-strand break repair pathway choice, EMBO J. 37 (2018) e100158, 10.15252/embj.2018100158. 30154076 PMC6138439

[118] Gao S, Feng S, Ning S, Liu J, Zhao H, Xu Y, Shang J, Li K, Li Q, Guo R, Xu D, An OB-fold complex controls the repair pathways for DNA double-strand breaks, Nat. Commun. 9 (2018) 3925, 10.1038/s41467-018-06407-7. 30254264 PMC6156606

[119] Boersma V, Moatti N, Segura-Bayona S, Peuscher MH, van der Torre J, Wevers BA, Orthwein A, Durocher D, Jacobs JJL, MAD2L2 controls DNA repair at telomeres and DNA breaks by inhibiting 5′ end resection, Nature 521 (2015) 537–540, 10.1038/nature14216. 25799990 PMC4481296

[120] King A, Reichl PI, Metson JS, Parker R, Munro D, Oliveira C, Sommerova L, Becker JR, Biggs D, Preece C, Davies B, Chapman JR, Shieldin and CST co-orchestrate DNA polymerase-dependent tailed-end joining reactions independently of 53BP1-governed repair pathway choice, Nat. Struct. Mol. Biol. (2024) 1–12, 10.1038/s41594-024-01381-9. 39227718 PMC11753365

[121] Mirman Z, Sasi NK, King A, Chapman JR, de Lange T, 53BP1–shieldin-dependent DSB processing in BRCA1-deficient cells requires CST–Polα_–_primase fill-in synthesis, Nat. Cell Biol. 24 (2022) 51–61, 10.1038/s41556-021-00812-9. 35027730 PMC8849574

[122] Paiano J, Zolnerowich N, Wu W, Pavani R, Wang C, Li H, Zheng L, Shen B, Sleckman BP, Chen B-R, Nussenzweig A, Role of 53BP1 in end protection and DNA synthesis at DNA breaks, Genes Dev. (2021), 10.1101/gad.348667.121. PMC8494207 34503990

[123] Susvirkar V, Faesen AC, Shieldin complex assembly kinetics and DNA binding by SHLD3, Commun. Biol. 6 (2023) 384, 10.1038/s42003-023-04757-7. 37031298 PMC10082759

[124] Rogers CM, Kaur H, Swift ML, Raina VB, Zhou S, Kawale AS, Syed S, Kelly KG, Jasper AM, Salunkhe S, Kwon Y, Wang J, Shabestari AB, Daley JM, Sacks A, Gaczynska ME, Osmulski PA, Rawal Y, Tomimatsu N, Gayther SA, Lawrenson K, Burma S, Wasmuth EV, Olsen SK, Zhao W, Hromas R, Libich DS, Mazin AV, Zhou D, Greene EC, Chowdhury D, Sung P, CTC1-STN1-TEN1 controls DNA break repair pathway choice via DNA end resection blockade, Science 388 (2025) 881–888, 10.1126/science.adt3034. 40403056 PMC12169213

[125] Zhao F, Kim W, Gao H, Liu C, Zhang Y, Chen Y, Deng M, Zhou Q, Huang J, Hu Q, Chen S-H, Nowsheen S, Kloeber JA, Qin B, Yin P, Tu X, Guo G, Qin S, Zhang C, Gao M, Luo K, Liu Y, Lou Z, Yuan J, ASTE1 promotes shieldin-complex-mediated DNA repair by attenuating end resection, Nat. Cell Biol. (2021) 1–11, 10.1038/s41556-021-00723-9. 34354233

[126] Tkáč J, Xu G, Adhikary H, Young JTF, Gallo D, Escribano-Díaz C, Krietsch J, Orthwein A, Munro M, Sol W, Al-Hakim A, Lin Z-Y, Jonkers J, Borst P, Brown GW, Gingras A-C, Rottenberg S, Masson J-Y, Durocher D, HELB Is a feedback inhibitor of DNA end resection, Mol. Cell 61 (2016) 405–418, 10.1016/j.molcel.2015.12.013. 26774285

[127] Hormeno S, Wilkinson OJ, Aicart-Ramos C, Kuppa S, Antony E, Dillingham MS, Moreno-Herrero F, Human HELB is a processive motor protein that catalyzes RPA clearance from single-stranded DNA, Proc. Natl. Acad. Sci. USA 119 (2022) e2112376119, 10.1073/pnas.2112376119. 35385349 PMC9169624

[128] Chen B-R, Sleckman BP, The regulation of DNA end resection by chromatin response to DNA double strand breaks, Front. Cell Dev. Biol. 10 (2022) 932633, 10.3389/fcell.2022.932633. 35912102 PMC9335370

[129] Adkins NL, Niu H, Sung P, Peterson CL, Nucleosome dynamics regulates DNA processing, Nat. Struct. Mol. Biol. 20 (2013) 836–842, 10.1038/nsmb.2585. 23728291 PMC3711194

[130] Costelloe T, Louge R, Tomimatsu N, Mukherjee B, Martini E, Khadaroo B, Dubois K, Wiegant WW, Thierry A, Burma S, van Attikum H, Llorente B, The yeast Fun30 and human SMARCAD1 chromatin remodellers promote DNA end resection, Nature 489 (2012) 581–584, 10.1038/nature11353. 22960744 PMC3493121

[131] Gospodinov A, Vaissiere T, Krastev DB, Legube G, Anachkova B, Herceg Z, Mammalian Ino80 mediates double-strand break repair through its role in DNA end strand resection, Mol. Cell. Biol. 31 (2011) 4735–4745, 10.1128/mcb.06182-11. 21947284 PMC3232918

[132] Hays E, Nettleton Elizabeth, Carter Caitlin, Morales Mariangel, Vo Lynn, Passo, Max R, Vélez-Cruz, The SWI/SNF ATPase BRG1 stimulates DNA end resection and homologous recombination by reducing nucleosome density at DNA double strand breaks and by promoting the recruitment of the CtIP nuclease, Cell Cycle 19 (2020) 3096–3114, 10.1080/15384101.2020.1831256. 33044911 PMC7714457

[133] Bantele SC, Ferreira P, Gritenaite D, Boos D, Pfander B, Targeting of the Fun30 nucleosome remodeller by the Dpb11 scaffold facilitates cell cycle-regulated DNA end resection, eLife 6 (2017) e21687, 10.7554/elife.21687. 28063255 PMC5300703

[134] Huang P-C, Hong S, Alnaser HF, Mimitou EP, Kim KP, Murakami H, Keeney S, Meiotic DNA break resection and recombination rely on chromatin remodeler Fun30, EMBO J. (2024) 1–25, 10.1038/s44318-024-00318-8. PMC11695836 39613969

[135] Densham RM, Garvin AJ, Stone HR, Strachan J, Baldock RA, Daza-Martin M, Fletcher A, Blair-Reid S, Beesley J, Johal B, Pearl LH, Neely R, Keep NH, Watts FZ, Morris JR, Human BRCA1–BARD1 ubiquitin ligase activity counteracts chromatin barriers to DNA resection, Nat. Struct. Mol. Biol. 23 (2016) 647–655, 10.1038/nsmb.3236. 27239795 PMC6522385

[136] Adkins NL, Swygert SG, Kaur P, Niu H, Grigoryev SA, Sung P, Wang H, Peterson CL, Nucleosome-like, single-stranded DNA (ssDNA)-histone octamer complexes and the implication for DNA double strand break repair, J. Biol. Chem. 292 (2017) 5271–5281, 10.1074/jbc.m117.776369. 28202543 PMC5392674

[137] Alatwi HE, Downs JA, Removal of H2A.Z by INO80 promotes homologous recombination, EMBO Rep. 16 (2015) 986–994, 10.15252/embr.201540330. 26142279 PMC4552491

[138] Sfeir A, de Lange T, Removal of shelterin reveals the telomere end-protection problem, Science 336 (2012) 593–597, 10.1126/science.1218498. 22556254 PMC3477646

[139] Kibe T, Zimmermann M, de Lange T, TPP1 blocks an ATR-mediated resection mechanism at telomeres, Mol. Cell 61 (2016) 236–246, 10.1016/j.molcel.2015.12.016. 26778124 PMC4724337

[140] Cai SW, de Lange T, CST–Polα/Primase: the second telomere maintenance machine, Genes Dev. (2023), 10.1101/gad.350479.123. PMC10499019 37495394

[141] Cai SW, Takai H, Zaug AJ, Dilgen TC, Cech TR, Walz T, de Lange T, POT1 recruits and regulates CST-Polα/primase at human telomeres, Cell (2024), 10.1016/j.cell.2024.05.002. PMC11246235 38838667

[142] He Q, Lin X, Chavez BL, Agrawal S, Lusk BL, Lim CJ, Structures of the human CST-Polα_–_primase complex bound to telomere templates, Nature 608 (2022) 826–832, 10.1038/s41586-022-05040-1. 35830881 PMC10268231

[143] Denchi EL, de Lange T, Protection of telomeres through independent control of ATM and ATR by TRF2 and POT1, Nature 448 (2007) 1068–1071, 10.1038/nature06065. 17687332

[144] Taylor AMR, Rothblum-Oviatt C, Ellis NA, Hickson ID, Meyer S, Crawford TO, Smogorzewska A, Pietrucha B, Weemaes C, Stewart GS, Chromosome instability syndromes, Nat. Rev. Dis. Prim. 5 (2019) 64, 10.1038/s41572-019-0113-0. 31537806 PMC10617425

[145] Maciejowski J, de Lange T, Telomeres in cancer: tumour suppression and genome instability, Nat. Rev. Mol. Cell Biol. 18 (2017) 175–186, 10.1038/nrm.2016.171. 28096526 PMC5589191

[146] Rossiello F, Jurk D, Passos JF, d’Adda di Fagagna F, Telomere dysfunction in ageing and age-related diseases, Nat. Cell Biol. 24 (2022) 135–147, 10.1038/s41556-022-00842-x. 35165420 PMC8985209

[147] Takai H, Jenkinson E, Kabir S, Babul-Hirji R, Najm-Tehrani N, Chitayat DA, Crow YJ, de Lange T, A POT1 mutation implicates defective telomere end fill-in and telomere truncations in Coats plus, Genes Dev. 30 (2016) 812–826, 10.1101/gad.276873.115. 27013236 PMC4826397

[148] Lin KY, Kraus WL, PARP inhibitors for cancer therapy, Cell 169 (2017) 183, 10.1016/j.cell.2017.03.034. 28388401

[149] D’Andrea AD, Mechanisms of PARP inhibitor sensitivity and resistance, DNA Repair 71 (2018) 172–176, 10.1016/j.dnarep.2018.08.021. 30177437

[150] Abramson J, Adler J, Dunger J, Evans R, Green T, Pritzel A, Ronneberger O, Willmore L, Ballard AJ, Bambrick J, Bodenstein SW, Evans DA, Hung C-C, O’Neill M, Reiman D, Tunyasuvunakool K, Wu Z, Žemgulytė A, Arvaniti E, Beattie C, Bertolli O, Bridgland A, Cherepanov A, Congreve M, Cowen-Rivers AI, Cowie A, Figurnov M, Fuchs FB, Gladman H, Jain R, Khan YA, Low CMR, Perlin K, Potapenko A, Savy P, Singh S, Stecula A, Thillaisundaram A, Tong C, Yakneen S, Zhong ED, Zielinski M, Žídek A, Bapst V, Kohli P, Jaderberg M, Hassabis D, Jumper JM, Accurate structure prediction of biomolecular interactions with AlphaFold 3, Nature 630 (2024) 493–500, 10.1038/s41586-024-07487-w. 38718835 PMC11168924

